# Differing growth responses to nutritional supplements in neighboring health districts of Burkina Faso are likely due to benefits of small-quantity lipid-based nutrient supplements (LNS)

**DOI:** 10.1371/journal.pone.0181770

**Published:** 2017-08-03

**Authors:** Sonja Y. Hess, Janet M. Peerson, Elodie Becquey, Souheila Abbeddou, Césaire T. Ouédraogo, Jérôme W. Somé, Elizabeth Yakes Jimenez, Jean-Bosco Ouédraogo, Stephen A. Vosti, Noël Rouamba, Kenneth H. Brown

**Affiliations:** 1 Program in International and Community Nutrition, Department of Nutrition, University of California Davis, Davis, CA, United States of America; 2 Poverty, Health and Nutrition Division, International Food Policy Research Institute, Washington, DC, United States of America; 3 Direction Régionale de l'Ouest, Institut de Recherche en Sciences de la Santé, Bobo-Dioulasso, Burkina Faso; 4 Center for Education Policy Research and Division of Epidemiology, Biostatistics and Preventive Medicine, Department of Internal Medicine, University of New Mexico, Albuquerque, NM, United States of America; 5 Department of Agricultural and Resource Economics, University of California, Davis, Davis, CA, United States of America; TNO, NETHERLANDS

## Abstract

**Background:**

Of two community-based trials among young children in neighboring health districts of Burkina Faso, one found that small-quantity lipid-based nutrient supplements (LNS) increased child growth compared with a non-intervention control group, but zinc supplementation did not in the second study.

**Objectives:**

We explored whether the disparate growth outcomes were associated with differences in intervention components, household demographic variables, and/or children’s morbidity.

**Methods:**

Children in the LNS study received 20g LNS daily containing different amounts of zinc (LNS). Children in the zinc supplementation study received different zinc supplementation regimens (Z-Suppl). Children in both studies were visited weekly for morbidity surveillance. Free malaria and diarrhea treatment was provided by the field worker in the LNS study, and by a village-based community-health worker in the zinc study. Anthropometric assessments were repeated every 13–16 weeks. For the present analyses, study intervals of the two studies were matched by child age and month of enrollment. The changes in length-for-age z-score (LAZ) per interval were compared between LNS and Z-Suppl groups using mixed model ANOVA or ANCOVA. Covariates were added to the model in blocks, and adjusted differences between group means were estimated.

**Results:**

Mean ages at enrollment of LNS (n = 1716) and Z-Suppl (n = 1720) were 9.4±0.4 and 10.1±2.7 months, respectively. The age-adjusted change in mean LAZ per interval declined less with LNS (-0.07±0.44) versus Z-Suppl (-0.21±0.43; p<0.0001). There was a significant group by interval interaction with the greatest difference found in 9–12 month old children (p<0.0001). Adjusting for demographic characteristics and morbidity did not reduce the observed differences by type of intervention, even though the morbidity burden was greater in the LNS group.

**Conclusions:**

Greater average physical growth in children who received LNS could not be explained by known cross-trial differences in baseline characteristics or morbidity burden, implying that the observed difference in growth response was partly due to LNS.

## Introduction

Linear growth restriction in early life continues to be a critical public health concern. Growth stunting is associated with increased mortality risk, impaired cognition and educational performance, lower adult wages, and, when accompanied by excessive weight gain later in childhood or adulthood, increased risk of nutrition-related chronic diseases [1]. The sustainable development goal 2.2 aims to end all forms of malnutrition by the year 2030, which includes achieving the global target to reduce the number of stunted children under 5 years of age by 40% by 2025 [2,3]. Although stunting has decreased slowly worldwide [4], achieving these goals poses a challenge. It is well-recognized that stunting is caused by many risk factors, including intrauterine growth restriction, inadequate breastfeeding and complementary feeding practices, household and family factors and repeated infection [5]. Thus, the expected impact and cost-effectiveness of nutritional interventions may vary according to these underlying risk factors.

The complementary feeding period, generally corresponding to the 6–24 month age range, is a particularly vulnerable period for growth faltering and therefore an important time to intervene. Small-quantity and medium-quantity lipid-based nutrient supplements (LNS) offer a promising strategy to improve the nutritional quality of local complementary foods [6]. Small-quantity LNS provide ~20 g or ~110–120 kcal per day and medium-quantity LNS ~50 g or ~250–280 kcal per day along with additional protein, essential fatty acids and 22 micronutrients [6]. While larger quantity LNS has proven useful in the treatment of children with moderate or severe acute malnutrition [7,8], the potential of smaller quantities of LNS to prevent malnutrition and increase growth is still being explored, with inconsistent effects on young children’s growth outcomes across studies in different settings [9–13]. One of the studies with a significant impact on children’s growth was conducted in south-western Burkina Faso. Children 9–18 months of age who received 20 g LNS daily along with illness treatment for malaria and diarrhea had significantly greater length and reduced stunting at 18 months of age compared to a non-intervention cohort [14]. Because the control group in this study was a non-intervention control group that did not receive small-quantity LNS, illness treatment or home visits, it is not clear how much of the growth impact was due to small-quantity LNS and/or illness treatment and how much was due to other aspects of the study design and study population characteristics. Thus, one of the key questions remaining is whether LNS was solely or partially responsible for the growth impact observed in Burkina Faso.

Another promising intervention to prevent childhood stunting is preventive zinc supplementation. Meta-analyses investigating the impact of preventive zinc supplementation found that 5–10 mg supplemental zinc daily has a small, but significant impact on linear growth and weight gain in prepubertal children [15–18]. This is in contrast to the recent findings among young children in Burkina Faso, where children 6–30 months of age who received daily preventive zinc supplementation, intermittent preventive zinc supplementation or therapeutic zinc supplementation gained slightly less length than children who did not receive any zinc supplements (3.15–3.20 cm vs. 3.36 cm over 16 weeks, p<0.001) [19]. The zinc supplementation study and the LNS study in Burkina Faso mentioned above were implemented in neighboring health districts using similar protocols, and therefore provide a unique opportunity for exploratory analyses of the reasons for these different growth outcomes. The purpose of the present paper is to assess whether the different growth outcomes were associated with differences in study design and form of supplementation, other aspects of the intervention design, demographic variables, or child morbidity in the two study populations.

## Material and methods

### Study design and procedures

Data from two studies are included in the present analyses. The LNS and the zinc supplementation studies were both partially masked, placebo-controlled, cluster-randomized intervention trials in neighboring health districts of southwestern Burkina Faso. The LNS trial was conducted from April 2010 to July 2012 in the Dandé Health District, and the zinc trial was conducted from December 2010 to February 2012 in the Orodara Health District. Ethical approvals for both study protocols and the consent procedures were issued by the Institutional Review Boards of the Centre Muraz in Bobo-Dioulasso (Burkina Faso) and the University of California, Davis (USA). The studies were registered as clinical trials with the U.S. National Institutes of Health (www.ClinicalTrials.gov; NCT00944281 and NCT00944359).

**Fig 1:**
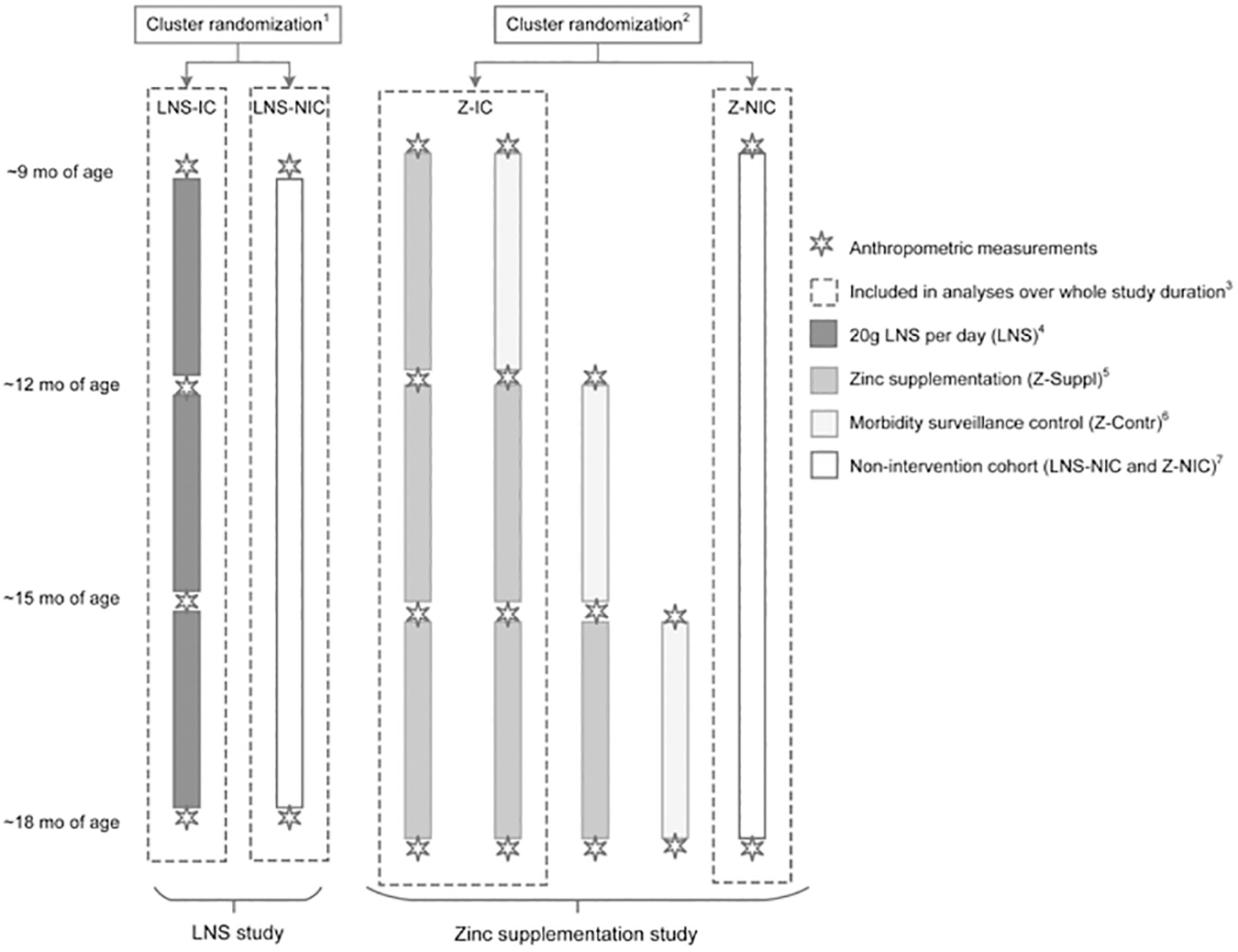
Study design of the LNS and the zinc supplementation studies. **Children eligible for the present long-term and short-term exploratory analyses were matched by age.** ^1^ In LNS study area, cluster randomization of 34 communities to intervention cohort (LNS-IC, 25 communities) or non-intervention cohort (LNS-NIC, 9 communities). ^2^ In zinc supplemenation study area, 36 geographically defined clusters, were randomly assigned to one of three cohorts: immediate and delayed intervention (Z-IC, 12 clusters each), and non-intervention cohort (Z-NIC, 12 clusters). ^3^ Children in LNS-IC, LNS-NIC, Z-IC, and Z-NIC who participated during the full study period were included in the analyses of the full study duration. ^4^ Within the LNS-IC communities, eligible children were randomly assigned to 1) LNS without zinc, and placebo tablet, 2) LNS with 5 mg zinc, and placebo tablet, 3) LNS with 10 mg zinc, and placebo tablet, or 4) LNS without zinc, and 5 mg zinc tablet. Children diagnosed with uncomplicated diarrhea, malaria and fever received free medical treatment during weekly home visit. ^5^ Within the Z-IC, eligible children were randomly assigned to 1) intermittent preventive zinc supplementation (10 mg zinc for 10 days) every 16 weeks and daily preventive and therapeutic placebo tablets, 2) daily preventive 7 mg zinc tablet daily and therapeutic placebo during diarrhea, and 3) therapeutic zinc supplementation for episodes of diarrhea (20 mg zinc/ day for 10 days) and daily placebo tablets. Children diagnosed with uncomplicated diarrhea, malaria and fever received free medical treatment from a village-based community health worker. ^6^ During each of the 16 week-rounds in the Z-IC cohort, three randomly selected clusters served as non-supplemented morbidity surveillance comparison group (Z-Contr). Children diagnosed with uncomplicated diarrhea, malaria and fever received free medical treatment from a village-based community health worker. ^4,5,6^ Children were matched by age and month of enrollment for inclusion in the analyses of age-specific intervals: 9–12 mo interval, 12–15 mo interval and 15–18 mo interval. ^7^ In both studies, children in the NIC were assessed at enrollment and at the end of the study and did not receive any supplementation of morbidity treatment throughout the course of the study.

Study procedures, participant characteristics and results of primary outcomes of both studies have been described in detail elsewhere [14,19] and are summarized briefly below. In both studies, communities were randomly allocated to intervention cohort (IC) or non-intervention cohort (NIC; Fig 1). In the intervention cohort of the zinc study (Z-IC), during each of the three intervals randomly selected clusters served as non-supplemented morbidity surveillance comparison group (Z-Contr) for the zinc supplemented groups (Z-Suppl). In both studies, children in the intervention communities were randomly assigned at the concession level (i.e. extended family compound) to different intervention products and supplementation schedules. Within the LNS-IC communities, eligible children were randomly assigned to receive a 20 g daily ration of LNS containing 0, 5 or 10 mg zinc [14]. Within the Z-IC, eligible children were randomly assigned to 1) intermittent preventive zinc supplementation (10 mg zinc for 10 days) every 16 weeks and daily preventive and therapeutic placebo tablets, 2) daily preventive 7 mg zinc tablet and therapeutic placebo during diarrhea, and 3) therapeutic zinc supplementation for episodes of diarrhea (20 mg zinc/ day for 10 days) and daily placebo tablets [19]. The LNS products and zinc and placebo tablets were produced by Nutriset SAS (Malaunay, France). The LNS for each treatment group were identical, except for their zinc content; and a daily LNS ration provided 118 kcal and 21 other micronutrients [6].

Children were eligible for the LNS study if they were 8.8 to 9.9 months of age, and for the zinc study if they were 6–27 months of age. Additional inclusion criteria were permanent residence in the study area, planned availability during the study period and written parental consent. Main exclusion criteria were: hemoglobin (Hb) <50 g/L, weight-for-length <70% of the median of the National Center for Health Statistics/World Health Organization (NCHS/WHO) growth reference [20], presence of bipedal edema, other severe illness warranting hospital referral, and congenital abnormalities potentially interfering with growth.

At enrollment, children’s length, weight and mid-upper arm circumference (MUAC) and maternal height and weight were measured. Information on children’s dietary practices [21] and household socio-economic status (SES) was obtained. A capillary blood sample was collected for analyses of hemoglobin (Hb) concentration (Hemocue 201+, HemoCue® AB, Ängelholm, Sweden) and a rapid diagnostic test (RDT) was performed for malaria parasites, based on histidine-rich protein II (SD BIOLINE Malaria Ag P.F/Pan, Standard Diagnostics, INC., Kyonggi-do, Korea). In case of illness, all children screened at enrollment for both studies received free medical treatment(s): oral rehydration salts (ORS) packets were provided for cases of diarrhea, and anti-malarial treatment was provided based on a positive RDT (artesunate-amodiaquine combination therapy in the LNS study and artemether-lumefantrine combination therapy in the zinc study), and paracetamol was provided to children with reported fever. Children with Hb <80 g/L received anthelmintic treatment (mebendazole 200 mg/day for 3 days) and iron supplements (2–6 mg iron/kg body weight for 30 days).

Anthropometric assessments were repeated every 12–13 weeks among children in the LNS-IC and every 16 weeks among children in the Z-IC (which includes Z-Suppl and Z-Contr). Length-for-age z-score (LAZ), weight-for-age z-score (WAZ), weight-for- length z-score (WLZ), and z-scores of body mass index (BMIZ) and mid-upper arm circumference (MUACZ) were calculated using the WHO growth standards [22,23]. A dedicated field worker visited homes of all children in the LNS-IC and Z-IC cohorts weekly to record morbidity symptoms for each of the past 7 days. At each visit, the field worker evaluated the child for the presence of clinical danger signs, and evidence of diarrhea, fever, or malaria. In case of reported fever on the day of or the day before the surveillance visit, the field worker performed an RDT and measured body temperature. Although the illness treatment protocol was comparable between the two studies, an important difference related to the site of treatment provision. In particular, for children in the LNS-IC, the field worker provided free treatment at the time of diagnosis during the home visit [14,24]. By contrast, in the Z-IC cohort of the zinc study (i.e. in the Z-Suppl and Z-Contr), children did not receive treatment directly from the field worker, but were encouraged to visit the project-trained community-health worker in their village of residence to receive free treatment [19]. Children with danger signs, or diarrhea, fever or malaria with complications and any other cases of severe illness were referred to the health center for evaluation and treatment in both studies. Children in LNS-NIC and Z-NIC had no further contact with the study team until the endline assessment and therefore were not measured at interim time points and did not receive intervention products, morbidity surveillance or free illness treatment.

### Statistical analyses

The objective of the analysis was to examine whether the different growth outcomes of the LNS and zinc trials could be explained by factors that differed between studies, other than the type of supplement. The general statistical approach was to estimate the difference between study effects using a linear model without covariates, and then assess whether this estimated difference was attenuated by including potential confounders in the model. If LNS had an independent positive effect on growth, as hypothesized, this difference would remain statistically significant, although possibly of reduced magnitude.

Two separate analyses were conducted: analysis of growth over the “whole study duration”, and analysis of growth over “age-specific study intervals”. Outcomes were change in LAZ, WAZ, and WLZ over the whole study duration and the age-specific intervals in relation to WHO growth standards [22].

**Fig 2:**
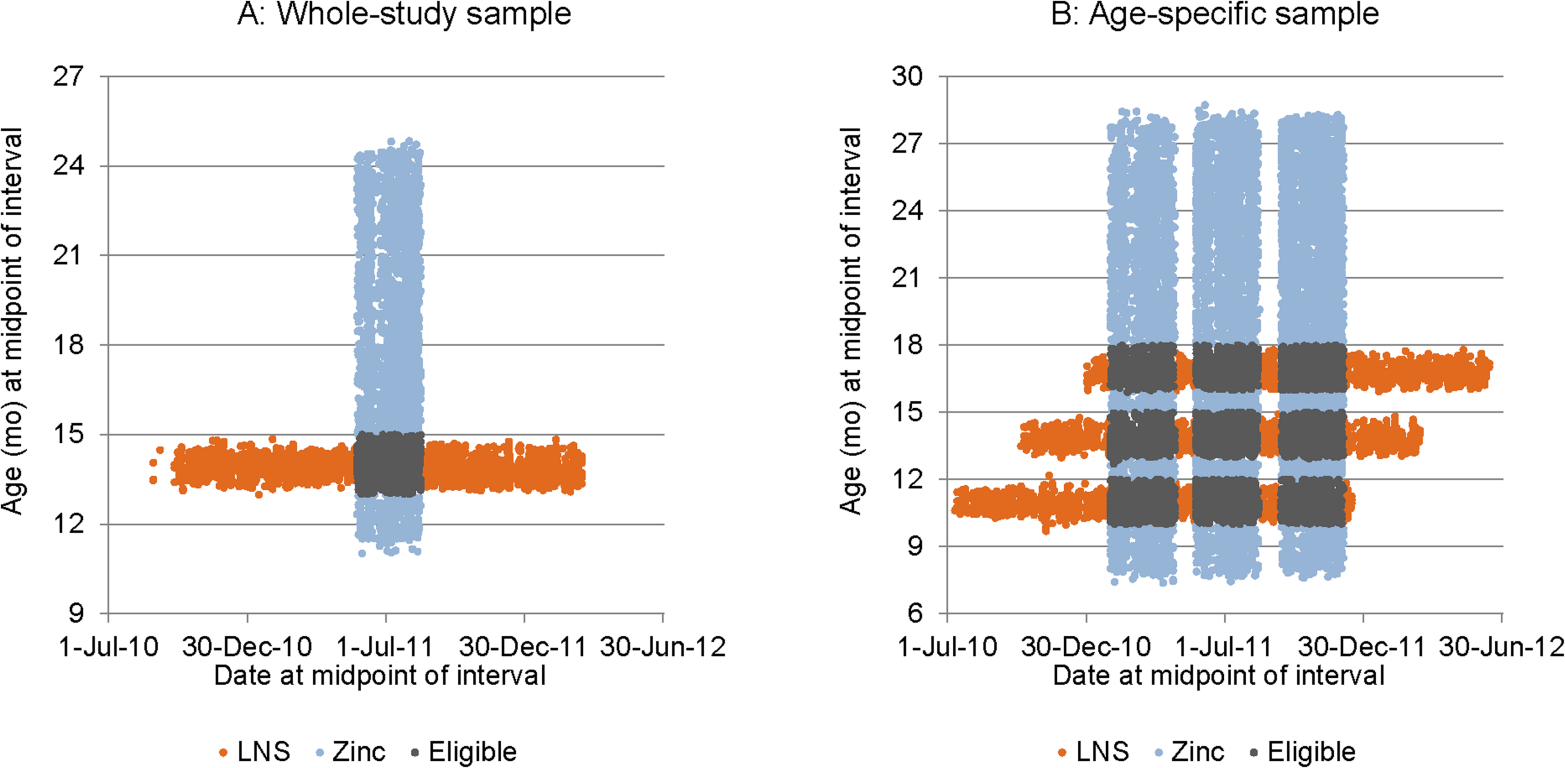
Age vs date at midpoint of study interval for whole-study and age-specific analyses of the LNS and the zinc supplementation study. Intervals in the overlapping areas were eligible for inclusion in the analysis.

Due to differences in the study design, all children in the LNS study, but only some of the children in the zinc supplementation study were included in the analyses over the whole study duration (Fig 1), which was approximately 39 weeks for the LNS study and 48 weeks for the zinc supplement study. Because the two studies included children of different ages and were implemented not completely at the same time, the following criteria were used to match participants of the two studies for the “whole study analysis”: children who had measurements at baseline and endline of study, for whom the date at midpoint could be matched between the two studies, and whose age at midpoint was between 13.0 and 15.0 months inclusive (Fig 2A). For the zinc supplementation study, outcomes were first compared between children who started with an initial non-supplemented morbidity surveillance period (Z-Contr) vs. those who started with supplementation (Z-Suppl); as there were no significant differences in growth outcomes over the full 48-week period, both groups of children were included in the analysis as Z-IC. Similarly, for the “age-specific analysis” inclusion criteria were: intervals for which there were measurements at the beginning and end, for which the dates at midpoint could be matched between the two studies, with child age at midpoint between 10.0 and 12.0 mo (9–12 mo interval) or 13.0 and 15.0 mo (12–15 mo interval) or 16.0 and 18.0 mo (15–18 mo interval), and for which the duration was between 2.1 and 4.3 mo (Fig 2B).

For both analyses, potential explanatory variables were identified as variables that differed between the two study samples, and therefore could be the source of the difference in the effect of treatment between studies. Covariates were added to the models in blocks (Table 1) for better understanding of the effect of each set of covariates. Morbidity covariates were not available for the whole-study analysis because the non-intervention cohort did not have morbidity measures.

**Table 1:** Components of mixed models used in the exploratory analyses of the whole study duration and the age-specific intervals of the LNS and the zinc supplementation study among young Burkinabe children.

Model number	Components of mixed model	Cohorts / study groups included
*Whole study duration*		
A	Fixed main effects of study (LNS *vs*. zinc study) and intervention cohort (IC *vs*. NIC), along with a fixed interaction term and random effects of village and concession within village	LNS-IC, LNS-NIC, Z-IC, Z-NIC
B	Model A + baseline anthropometric values (LAZ, WLZ), age at mid-point and time between anthropometric measurements	LNS-IC, LNS-NIC, Z-IC, Z-NIC
C	Model B + sex of child, maternal height, BMI, age, marital status (first wife), ethnic group, maternal education	LNS-IC, LNS-NIC, Z-IC, Z-NIC
*Age-specific interval*		
1	Fixed effect of group and random effects of village and concession within village	LNS, Z-Suppl, Z-Contr
2	Model 1 + duration of interval (i.e. time between two anthropometric measurements)	LNS, Z-Suppl, Z-Contr
3	Model 2 + age at midpoint of interval and season	LNS, Z-Suppl, Z-Contr
4	Model 3 + child sex, maternal age, height, BMI, educational level, marital status, and ethnic group, and anthropometric status at enrollment	LNS, Z-Suppl, Z-Contr
5	Model 4 + prevalence of fever without malaria, prevalence of fever with malaria, incidence of treated malaria, prevalence of diarrhea, incidence of severe diarrhea, and prevalence of upper or lower respiratory infection	LNS, Z-Suppl, Z-Contr
6	Model 5 + anthropometric variables at the beginning of the age-specific interval (potentially mediating variables)	LNS, Z-Suppl, Z-Contr

For the whole-study analysis, the following basic variance components model was fit:

$$Y_{ijklm}=\beta_{0}+\beta_{1}S_{i}+\beta_{2}C_{j}+\beta_{3}S_{i}C_{j}+\gamma_{k}+\delta_{l\left(k\right)}+\varepsilon_{ijklm}$$

where: *Y*_*ijklm*_ = Outcome (change in LAZ, WAZ, or WLZ) for child *m* in study *i*, cohort *j*, village *k*, and concession *l*

*S*_*i*_ = Indicator variable for study (LNS vs Zinc)
*C*_*j*_ = Indicator variable for cohort (IC vs NIC)
*γ*_*k*_ = Random effect for village *k*
*δ*_*l(k)*_ = Random effect for concession *l* nested within village k
*ε*_*ijklm*_ = Error term for child *m*

The coefficient for the study by cohort interaction, β_3_, estimates the difference between treatment effects in the two studies. Potential confounder variables, along with their interaction with cohort, were then added to the model in stages:

$$Y_{ijklm}=\beta_{0}^{'}+\beta_{1}^{'}S_{i}+\beta_{2}^{'}C_{j}+\beta_{3}^{'}S_{i}C_{j}+\underset{g}{\sum }\left(\theta_{g}X_{g,ijklm}+\varphi_{g}C_{j}X_{g,ijklm}\right)+\gamma_{k}^{'}+\delta_{l\left(k\right)}^{'}+\varepsilon_{ijklm}^{'}$$

where: *X*_*g*,*ijklm*_ = Value of covariate g for child *m* in study *i*, cohort *j*, village *k*, and concession *l*, standardized to a mean of 0

The new coefficient for the study by cohort interaction, β_3_^’^, estimates the theoretical difference between treatment effects in the two studies at the mean value of the covariates.

The analysis of age-specific intervals was carried out to better understand both the difference in treatment effects at different ages and the effect of morbidity variables on the treatment differential. Individual intervals were designated according to the treatment received (LNS, Z-Suppl, Z-Contr) in that interval. Because the NIC groups could not be included, due to not having age-specific data, and there was no LNS control interval, the effects of the different study interventions was examined by comparing the LNS and Z-Suppl groups, with the Z-Contr group included in the analysis for informal comparison. A preliminary mixed models analysis including fixed effects of group (LNS, Z-Suppl, Z-Contr), age interval (9–12 mo, 12–15 mo, 15–18 mo), and group by age interval, and random effects of village, concession, and subject, indicated that in general the group by age interval interaction was significant and random effects of subject were minimal. Therefore, the analysis was conducted separately for each age-specific interval, with the following basic variance components model:

$$Y_{iklm}=\beta_{0}+\beta_{1}G_{1i}+\beta_{2}G_{2i}+\gamma_{k}+\delta_{l\left(k\right)}+\varepsilon_{ijklm}$$

where: *Y*_*ijklm*_ = Outcome (change in LAZ, WAZ, or WLZ) for child *m* in group *i*, village *k*, and concession *l*

*G*_*1i*_ = Indicator variable for LNS vs Z-Suppl
*G*_*2i*_ = Indicator variable for Z-Contr vs Z-Suppl
*γ*_*k*_ = Random effect for village *k*
*δ*_*l(k)*_ = Random effect for concession *l* nested within village k
*ε*_*ijklm*_ = Error term for child *m*

The coefficient for the indicator variable for LNS vs Z-Suppl, β_1_, estimates the difference between the LNS and the Z-Suppl group means. Confounder variables were then added to the model in stages:

$$Y_{ijklm}=\beta_{0}^{'}+\beta_{1}^{'}G_{1i}+\beta_{2}^{'}G_{2i}+\underset{g}{\sum }\left(\theta_{g}X_{g,ijklm}\right)+\gamma_{k}^{'}+\delta_{l\left(k\right)}^{'}+\varepsilon_{ijklm}^{'}$$

where: *X*_*g*,*ijklm*_ = Value of covariate g for child *m* in group *i*, village *k*, and concession *l*, optionally standardized to a mean of 0

The new coefficient for the indicator variable for LNS vs Z-Suppl, β1’, estimates the theoretical difference between the groups at the mean value of the covariates. Analyses were conducted with the MIXED procedure in SAS for Windows, Release 9.4 (Cary, NC).

## Results

Separate sets of analyses were completed for growth increments during the whole study duration and for age-specific intervals, so each set of results is reported separately below.

### Whole study analysis

Baseline characteristics differed between studies (Table 2). Namely, children in the LNS study were significantly older at enrollment than children in the zinc supplementation study (9.5 ± 0.4 months vs. 8.5 ± 0.6, <0.0001), and children in the LNS study tended to have lower mean LAZ, WAZ, WLZ, and MUACZ. However, only baseline length and baseline LAZ remained significantly different between studies after controlling for age. Mothers in the LNS study were slightly older and taller, but had a lower BMI, and mothers in the zinc supplementation study were more likely to have had any formal education. There were also some differences in mothers’ marital status, religion and ethnicity. Based on these baseline comparisons between studies, differences in the study growth outcomes may in part be explained by differences in baseline LAZ and baseline age of the children and maternal characteristics such as maternal education, marital status, religion and ethnic group. Thus, to explore the change in growth outcomes over the whole study period, we added those covariates that differed significantly at baseline to the model, as described below.

**Table 2:** Comparison of baseline characteristics of children in the exploratory analyses covering the whole duration of the intervention trials.^1^

					p-value^3^			p-value after controlling for age at baseline^3^		
	LNS-IC	LNS-NIC	Z-IC^2^	Z-NIC	study	Intervention cohort	study* intervention cohort	study	Intervention cohort	study* intervention cohort
N	402	129	261	207						
Study duration (wks)	38.4 ± 0.9	39.3 ± 0.7	48.2 ± 0.7	48.1 ± 0.8	<0.0001	0.012	0.012	n/a	n/a	n/a
**Child characteristics at enrollment**										
Age (mo)	9.5 ± 0.4	9.5 ± 0.4	8.5 ± 0.6	8.5 ± 0.6	<0.0001	0.98	0.35	n/a	n/a	n/a
Male, n (%)	197 (49.0)	58 (45.0)	130 (49.8)	88 (42.5)	0.81	0.10	0.63	n/a	n/a	n/a
Hemoglobin (g/L)	90.7 ± 15.6	90.0 ± 15.4	92.1 ± 15.1	92.5 ± 14.8	0.22	0.85	0.99	0.70	0.85	0.97
Length (cm)	68.7 ± 2.5	68.7 ± 2.4	68.0 ± 2.8	68.3 ± 2.8	0.005	0.37	0.63	0.047	0.36	0.47
Weight (kg)	7.4 ± 0.9	7.4 ± 1.0	7.3 ± 1.0	7.4 ± 1.0	0.99	0.36	0.35	0.13	0.36	0.31
MUAC (cm)	13.2 ± 1.1	13.1 ± 1.1	13.3 ± 1.2	13.5 ± 1.1	0.046	0.46	0.67	0.38	0.47	0.70
LAZ	-1.28 ± 1.04	-1.24 ± 0.88	-1.04 ± 1.15	-0.83 ± 1.12	0.0001	0.13	0.34	0.045	0.14	0.37
WAZ	-1.47 ± 1.05	-1.49 ± 1.11	-1.33 ±1.19	-1.08 ± 1.06	0.001	0.18	0.15	0.11	0.18	0.17
WLZ	-1.01 ± 0.98	-1.09 ± 1.12	-0.95 ± 1.07	-0.75 ± 0.97	0.008	0.48	0.13	0.41	0.48	0.14
BMIZ	-1.00 ± 0.98	-1.07 ± 1.12	-1.00 ± 1.08	-0.81 ± 0.98	0.076	0.51	0.15	0.48	0.51	0.16
MUACZ	-1.05 ± 1.07	-1.09 ± 1.05	-0.88 ± 1.19	-0.68 ± 1.02	0.010	0.35	0.51	0.36	0.35	0.56
Breastfeeding in past 24hrs, n (%)	402 (100.0)	129 (100.0)	259 (99.6)	206 (99.5)	n/a	n/a	n/a	n/a	n/a	n/a
Minimum dietary diversity, n(%)^4^	36 (9.0)	10 (7.8)	22 (8.7)	14 (6.8)	0.73	0.41	0.82	0.95	0.41	0.83
**Maternal characteristics**										
Maternal age (yrs)	27.8 ± 7.1	27.4 ± 5.7	25.8 ± 6.3	26.4 ± 6.4	0.002	0.78	0.25	n/a	n/a	n/a
Maternal height (cm)	162.1 ± 5.8	162.0 ± 5.2	160.1 ± 6.1	161.6 ± 5.9	0.011	0.10	0.066	n/a	n/a	n/a
Maternal BMI (kg/m^2^)	20.8 ± 2.6	20.8 ± 1.9	21.2 ± 2.4	21.8 ± 3.1	0.0008	0.13	0.22	n/a	n/a	n/a
Maternal education, n (% with any formal education)	44 (11.0)	9 (7.0)	57 (22.0)	45 (22.7)	<0.0001	0.41	0.29	n/a	n/a	n/a
Mother is first or only wife, n (%)	289 (72.3)	91 (70.5)	156 (60.2)	118 (59.6)	0.003	0.72	0.94	n/a	n/a	n/a
Maternal religion					0.045	0.93	0.11	n/a	n/a	n/a
Muslim, n (%)	317 (79.1)	85 (65.9)	202 (79.2)	155 (79.1)						
Traditional, n (%)	42 (10.5)	39 (30.2)	46 (18.0)	26 (13.3)						
Christian, n (%)	42 (10.5)	5 (3.9)	7 (2.8)	15 (7.7)						

BMI, body mass index; BMIZ, z-score of body mass index; LAZ, length-for-age z-score; MUAC, mid-upper arm circumference; MUACZ; z-score of mid-upper arm circumference; WAZ, weight-for-age z-score; WLZ, weight-for-length z-score

^1^ mean ± standard deviation (SD), and n (%); all such values.

^2^Some of the children in this sample participated in morbidity surveillance first for 16 weeks and subsequently received zinc supplements

^3^Comparison of baseline characteristics with mixed models ANOVA for continuous outcomes and mixed models logistic regression for categorical outcomes, including random effects of village and concession.

^4^ Child received food from 4 or more food groups during the previous 24 h [21]

**Fig 3:**
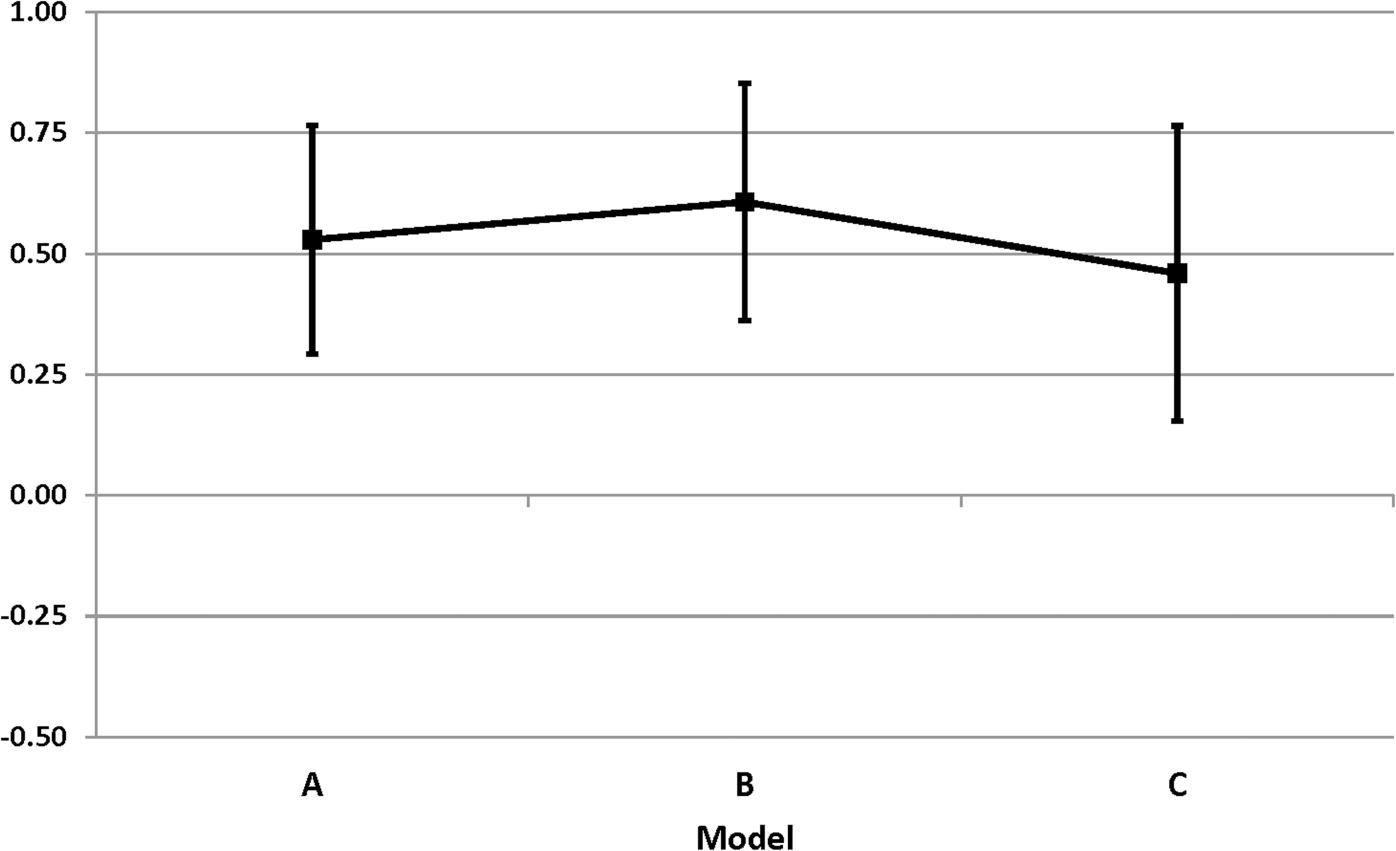
Difference in change in length-for-age z-score between the LNS and the zinc study populations over the whole study duration.

Adding initial LAZ, initial WLZ, child’s age at mid-point and time between length measurements did not attenuate the study by cohort interaction coefficient for change in LAZ (model B; 0.607 ± 0.123) compared with the simple model unadjusted for any covariates (model A; 0.529 ± 0.119; Fig 3). Similarly, the addition of maternal and child covariates that differed at baseline, did not substantially attenuate the interaction coefficient (model C; 0.459 ± 0.152), suggesting that the differences in final LAZ were not explained by these covariates (Table 3). These findings were similar for WAZ. When exploring the change in WLZ, we found that there was no significant study by cohort interaction term in the unadjusted model A. After the addition of covariates, the interaction term became significant, suggesting that not just height, but also weight-for-height was affected by differences between the studies other than the covariates, indicating that differences may be due to the provision of LNS.

**Table 3:** Comparison of the change in length-for-age z-score, weight-for-age z-score, and weight-for-length z-score between the LNS and the zinc study populations over the whole study duration.^1^

	Adjusted means				Interaction coefficient	p-value		
Model	LNS-IC	LNS-NIC	Z-IC	Z-NIC		Study	Intervention	Study*intervention
**Change in LAZ**								
A: No covariates^2^	-0.146 ± 0.047	-0.550 ± 0.076	-0.676 ± 0.050	-0.551 ± 0.060	0.529 ± 0.119	< .0001	0.022	< .0001
B: Initial LAZ, initial WLZ, age at midpoint, time between measurements (covariate only)	-0.044 ± 0.129	-0.475 ± 0.126	-0.797 ± 0.146	-0.622 ± 0.148	0.607 ± 0.123	0.080	0.038	< .0001
C: Above plus child sex and maternal covariates^3^	-0.095 ± 0.127	-0.483 ± 0.124	-0.729 ± 0.155	-0.657 ± 0.156	0.459 ± 0.152	0.120	0.012	0.004
**Change in WAZ**								
A: No covariates^2^	0.212 ± 0.037	-0.103 ± 0.065	-0.185 ± 0.047	-0.124 ± 0.053	0.376 ± 0.103	0.0001	0.017	0.0005
B: Initial WAZ, age at midpoint, time between measurements (covariate only)	0.366 ± 0.125	-0.003 ± 0.115	-0.382 ± 0.144	-0.232 ± 0.142	0.519 ± 0.097	0.055	0.023	< .0001
C: Above plus child sex and maternal covariates^3^	0.355 ± 0.125	-0.014 ± 0.115	-0.362 ± 0.154	-0.283 ± 0.151	0.447 ± 0.132	0.058	0.005	0.002
**Change in WLZ**								
A: No covariates^2^	0.232 ± 0.044	0.090 ± 0.077	0.030 ± 0.054	0.035 ± 0.062	0.147 ± 0.121	0.036	0.263	0.226
B: Initial LAZ, initial WLZ, age at midpoint, time between measurements (covariate only)	0.475 ± 0.135	0.232 ± 0.125	-0.262 ± 0.156	-0.184 ± 0.154	0.321 ± 0.105	0.037	0.109	0.003
C: Above plus maternal and child covariates^3^	0.443 ± 0.133	0.187 ± 0.123	-0.245 ± 0.165	-0.191 ± 0.161	0.310 ± 0.139	0.056	0.050	0.031

LAZ, length-for-age z-score; WAZ, weight-for-age z-score; WLZ, weight-for-length z-score

^1^adjusted mean ± SE

^2^Fixed main effect of study (LNS vs. zinc study) and intervention (IC vs NIC), along with fixed interaction term and random effects of village and concession within village

^3^Maternal covariates included in the model were: height, BMI, age, marital status (first wife), ethnic group, maternal education

### Age-specific analysis

As mentioned previously, anthropometric assessments were completed only at the beginning and end of the study in the non-intervention groups, and these groups were not included in the morbidity surveillance. Thus, results for age-specific intervals and for morbidity surveillance are available only for the intervention cohorts (Fig 1).

The sample size per study group and age range included in the present analyses ranged from 185 to 221 in the Z-Contr group and 769 to 975 in the two supplemented groups (Z-Suppl and LNS) during the different study rounds (Table 4). Children in the Z-Contr group were significantly older (12.3 ± 2.4 mo) than children in the Z-Suppl group (10.1 ± 2.7 mo) and the LNS-IC group (9.4 ± 0.4 mo; p<0.0001). However, baseline LAZ, WAZ and WLZ were not significantly different among the 3 groups with or without adjustment for age (Table 5). Maternal age, height, BMI, education, marital status and ethnic group differed significantly among the 3 groups. The morbidity burden was highest in the LNS group (Table 4). In particular, malaria prevalence and incidence were significantly higher in the LNS group compared to the other two study groups. Although treatment coverage differed between the two studies, it was high in all study groups. In particular, 99.5% of all identified malaria cases were treated in the LNS group, 90.1% in the Z-Suppl group and 94.8% in the Z-Contr group (P<0.0001). Diarrhea prevalence tended to be higher in the LNS and the Z-Contr groups compared to the Z-Suppl group (Table 4) and 38.7% of all diarrhea cases were treated in the LNS group compared to 30.0% in the Z-Contr and 29.2% in the Z-Suppl groups, respectively (P<0.0001). In summary, child age at enrollment as well as several maternal characteristics and the morbidity burden were identified as potential confounders and these were included in the model described below.

**Table 4:** Prevalence of malaria, fever and diarrhea during the different age intervals.

	Age group	LNS	Z-Contr	Z-Suppl	P-value
N^1^	9–12 mo	975	183	768	
	12–15 mo	827	220	803	
	15–18 mo	793	203	881	
Malaria prevalence (%)	9–12 mo	1.30 (0.07) ^a^	0.57 (0.10) ^b^	0.60 (0.04) ^b^	<0.0001
	12–15 mo	1.60 (0.09) ^a^	0.69 (0.09) ^b^	0.49 (0.04) ^c^	<0.0001
	15–18 mo	1.36 (0.08) ^a^	0.80 (0.09) ^b^	0.54 (0.03) ^c^	<0.0001
Fever prevalence (%)	9–12 mo	3.34 (0.12) ^a^	2.98 (0.22) ^a^	2.24 (0.10) ^b^	<0.0001
	12–15 mo	3.27 (0.13) ^a^	3.31 (0.21) ^a^	2.00 (0.09) ^b^	<0.0001
	15–18 mo	3.02 (0.13) ^a^	3.20 (0.25) ^a^	1.79 (0.07) ^**b**^	<0.0001
Diarrhea prevalence (%)	9–12 mo	3.24 (0.15) ^a^	2.08 (0.22) ^b^	2.15 (0.11) ^b^	<0.0001
	12–15 mo	2.78 (0.15) ^a^	2.24 (0.21) ^ab^	1.79 (0.10) ^b^	<0.0001
	15–18 mo	2.26 (0.14) ^a^	2.16 (0.30) ^a^	1.36 (0.09) ^b^	<0.0001
Acute upper respiratory infection prevalence (%)	9–12 mo	7.48 (0.49) ^a^	1.92 (0.40) ^b^	1.34 (0.14) ^b^	<0.0001
	12–15 mo	6.91 (0.44) ^a^	2.06 (0.34) ^b^	1.26 (0.12) ^c^	<0.0001
	15–18 mo	7.17 (0.48) ^a^	2.50 (0.40) ^b^	1.23 (0.10) ^c^	<0.0001
Acute lower respiratory infection prevalence (%)	9–12 mo	0.11 (0.05)	0.07 (0.03)	0.09 (0.02)	0.856
	12–15 mo	0.05 (0.02)	0.04 (0.03)	0.04 (0.01)	0.882
	15–18 mo	0.05 (0.02)	0.08 (0.03)	0.04 (0.01)	0.562

Prevalences were defined as number of days with illness per 100 days of observation, and were compared between groups with mixed models Poisson regression (SAS GENMOD procedure), with overdispersion adjustment. Rows with different superscripts differ significantly (p<0.05)

^1^ Sample size for different morbidity outcomes may vary slightly.

**Table 5:** Comparison of baseline characteristics of children in the exploratory analyses of the LNS and zinc intervention trials by age range.^1^

	LNS	Z-Contr	Z-Suppl	p-value	p-value after controlling for age at enrollment
N	1716	610	1720		
Child characteristics					
Age (mo)	9.4 ± 0.4	12.3 ± 2.4	10.1 ± 2.7	<0.0001	n/a
Male	877 (51.1)	302 (49.5)	882 (51.3)	0.74	n/a
Hemoglobin (g/L)	89.8 ± 15.5	90.4 ± 14.1	92.3 ± 14.6	0.041	0.049
Length (cm)	68.8 ± 2.6	71.6 ± 3.6	69.5 ± 4.1	<0.0001	0.30
Weight (kg)	7.42 ± 0.98	7.93 ± 1.11	7.58 ± 1.16	<0.0001	0.44
MUAC (cm)	13.32 ± 1.16	13.47 ± 1.11	13.45 ± 1.20	0.08	0.07
LAZ	-1.22 ± 1.09	-1.38 ± 1.20	-1.18 ± 1.19	0.12	0.11
WAZ	-1.43 ± 1.12	-1.48 ± 1.15	-1.36 ± 1.18	0.22	0.17
WLZ	-1.00 ± 1.05	-1.06 ± 1.00	-0.95 ± 1.07	0.066	0.26
BMIZ	-0.99 ± 1.05	-0.92 ± 0.98	-0.93 ± 1.07	0.30	0.35
MUACZ	-0.95 ± 1.09	-0.91 ± 1.04	-0.84 ± 1.12	0.030	0.039
Breastfeeding in past 24hrs, n (%)	1715 (99.9)	586 (99.8)	1663 (99.5)	0.085	0.095
Minimum dietary diversity, n(%)^2^	208 (12.1)	144 (26.7)	242 (15.2)	<0.0001	0.93
**Maternal characteristics**					
Maternal age (yrs)	27.1 ± 6.9	25.8 ± 6.7	26.1 ± 6.4	<0.0001	n/a
Maternal height (cm)	162.3 ± 5.7	160.1 ± 5.7	159.8 ± 6.1	<0.0001	n/a
Maternal BMI (kg/m^2^)	20.9 ± 2.5	21.2 ± 2.5	21.3 ± 2.5	0.003	n/a
Maternal education, n (% with any formal education)	185 (10.9)	113 (19.0)	330 (19.7)	0.003	n/a
Mother is first or only wife, n (%)	1251 (73.3)	346 (58.2)	965 (57.7)	<0.0001	n/a
Maternal religion				0.49	n/a
Muslim, n (%)	1412 (82.8)	471 (80.1)	1276 (78.3)		
Traditional, n (%)	160 (9.4)	75 (12.8)	264 (16.2)		
Christian, n (%)	134 (7.9)	38 (6.5)	89 (5.5)		

BMI, body mass index; BMIZ, z-score of body mass index; LAZ, length-for-age z-score; MUAC, mid-upper arm circumference; MUACZ; z-score of mid-upper arm circumference; WAZ, weight-for-age z-score; WLZ, weight-for-length z-score

^1^ mean ± SD, and n (%); all such values.

^2^ Child received food from 4 or more food groups during the previous 24 h [21]

**Fig 4:**
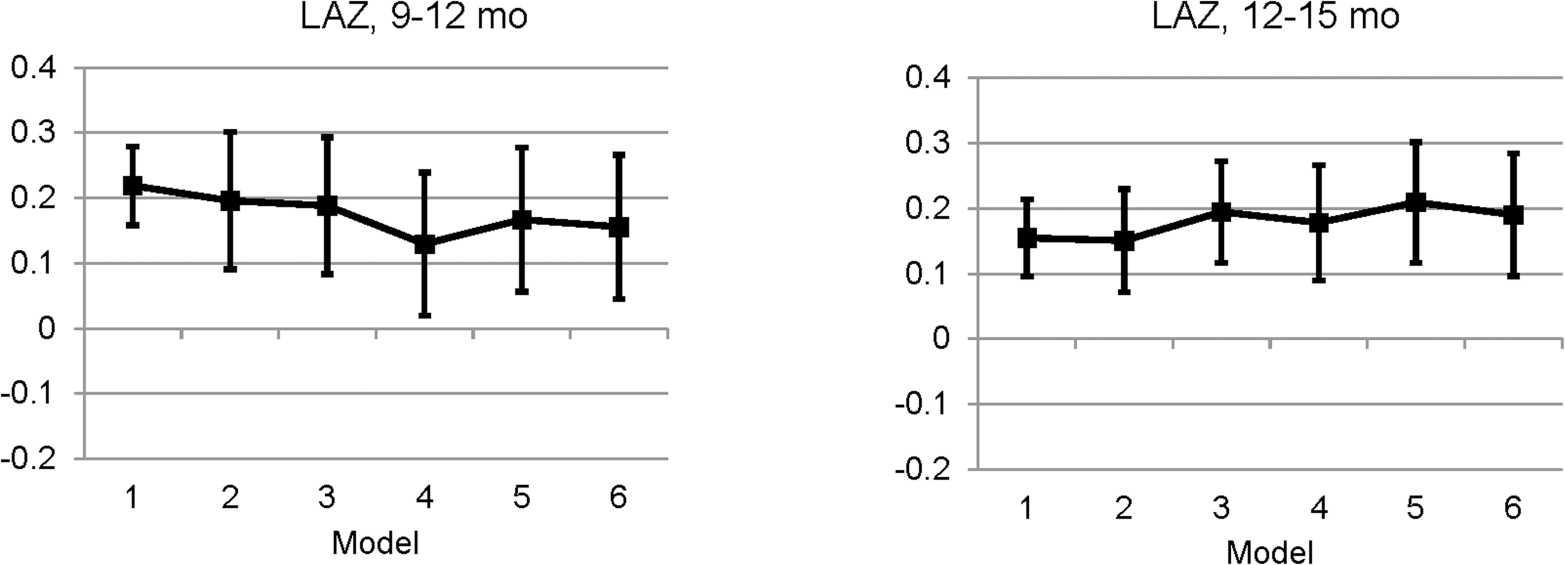
The difference in mean changes in length-for-age z-score between the LNS and the Z-Suppl groups from 9 to 12 and 12 to 15 months of age as different covariates are added to the mixed-model analysis of covariance.

In Table 6 we show the effect of adding covariates to the model on the differences in mean changes of LAZ, WAZ and WLZ from 9 to 12 months of age. Unadjusted change in LAZ from 9 to 12 months of age was significantly different between LNS and Z-Suppl and between LNS and Z-Contr, but not between Z-Suppl and Z-Contr. Adding the time between the anthropometric assessments (model 1) along with child age and seasonality (model 2) attenuates the difference in mean change between LNS and Z-Suppl and between LNS and Z-Contr somewhat, but the differences remain significant. The differences disappear between LNS and Z-Contr as enrollment covariates are added to the model, but remain marginally significant between LNS and Z-Suppl (model 4). Adding the morbidity prevalence and incidence during the specific age interval to the model increases the difference in mean changes of LAZ between LNS and Z-Suppl, implying that the morbidity burden and treatment provided did not explain the difference in growth impact observed between these groups with previous models. Similarly, adding covariates to the model does not explain the differences in mean changes of WAZ. The differences in mean changes in WAZ remain significant between LNS and Z-Suppl and LNS and Z-Contr after adding baseline characteristics, morbidity burden and baseline anthropometric Z-scores. While this is also true for the differences in mean changes in WLZ between LNS and Z-Contr, the differences in mean changes in WLZ between LNS and Z-Suppl are no longer significant in the adjusted models. Table 7 shows that differences in mean change in LAZ from 12 to 15 months of age remain significantly different between LNS and Z-Suppl and between LNS and Z-Contr throughout the increasingly adjusted model, implying that the impact found on growth in the LNS group could not be explained by any of the covariates tested. The unadjusted group differences were not significant for mean changes of WAZ and WLZ from 12 to 15 months, nor for mean change in LAZ, WAZ and WLZ from 15 to 18 months. Thus those models are not shown. The differences in mean change in LAZ from 9 to 12 months and 12 to 15 months of age between LNS and Z-Suppl are shown in Fig 4.

**Table 6:** The change and the difference in mean changes of length-for-age z-score, weight-for-age z-score and weight-for-length z-score from 9 to 12 months of age as different covariates are added to the mixed-model analysis of covariance.

Model	Adjusted means of LAZ			Difference in mean changes of LAZ			P-values for differences		
	Z-Contr	Z-Suppl	LNS	Z-Suppl *vs* Z-Contr	LNS *vs* Z-Contr	LNS *vs* Z-Suppl	Z-Suppl *vs* Z-Contr	LNS *vs* Z-Contr	LNS *vs* Z-Suppl
Change in LAZ from 9–12 months									
1: No covariates	-0.308 (0.036)	-0.307 (0.021)	-0.088 (0.022)	0.001	0.22	0.219	0.100	< .0001	< .0001
2: Time between measurements only	-0.297 (0.042)	-0.295 (0.031)	-0.099 (0.031)	0.002	0.198	0.196	0.999	0.003	0.001
3: Time between measurements, age at midpoint, season	-0.283 (0.043)	-0.293 (0.031)	-0.105 (0.031)	-0.01	0.178	0.188	0.965	0.010	0.001
4: Above plus covariates at enrollment	-0.240 (0.043)	-0.267 (0.033)	-0.138 (0.030)	-0.028	0.102	0.13	0.783	0.213	0.055
5: Above plus morbidity prevalence and incidence	-0.260 (0.043)	-0.286 (0.033)	-0.119 (0.030)	-0.027	0.14	0.167	0.793	0.057	0.009
6: Above plus LAZ, WAZ and WLZ at start of age-specific period	-0.255 (0.042)	-0.279 (0.033)	-0.124 (0.030)	-0.024	0.132	0.156	0.829	0.078	0.016
Change in WAZ from 9–12 months									
1: No covariates	-0.150 (0.040)	-0.103 (0.019)	0.157 (0.017)	0.047	0.307	0.26	0.536	< .0001	< .0001
2: Time between measurements only	-0.140 (0.047)	-0.092 (0.032)	0.147 (0.030)	0.047	0.286	0.239	0.531	< .0001	0.0001
3: Time between measurements, age at midpoint, season	-0.132 (0.047)	-0.088 (0.033)	0.142 (0.031)	0.044	0.274	0.23	0.580	0.0002	0.0002
4: Above plus covariates at enrollment	-0.099 (0.047)	-0.039 (0.035)	0.099 (0.030)	0.059	0.197	0.138	0.387	0.009	0.058
5: Above plus morbidity prevalence and incidence	-0.114 (0.047)	-0.057 (0.035)	0.113 (0.030)	0.056	0.226	0.17	0.421	0.002	0.014
6: Above plus LAZ, WAZ and WLZ at start of age-specific period	-0.115 (0.047)	-0.059 (0.035)	0.114 (0.030)	0.056	0.228	0.172	0.419	0.002	0.013
Change in WLZ from 9–12 months									
1: No covariates	-0.150 (0.050)	-0.068 (0.026)	0.144 (0.025)	0.082	0.294	0.212	0.3021	< .0001	< .0001
2: Time between measurements only	-0.125 (0.059)	-0.042 (0.042)	0.119 (0.040)	0.083	0.245	0.161	0.2949	0.01	0.0716
3: Time between measurements, age at midpoint, season	-0.124 (0.059)	-0.040 (0.041)	0.116 (0.040)	0.084	0.24	0.156	0.288	0.012	0.0819
4: Above plus covariates at enrollment	-0.118 (0.056)	-0.008 (0.042)	0.085 (0.036)	0.11	0.204	0.094	0.1008	0.0276	0.3919
5: Above plus morbidity prevalence and incidence	-0.126 (0.056)	-0.021 (0.042)	0.093 (0.036)	0.104	0.219	0.114	0.1253	0.0166	0.2506
6: Above plus LAZ, WAZ and WLZ at start of age-specific period	-0.130 (0.056)	-0.024 (0.042)	0.096 (0.036)	0.106	0.225	0.12	0.1175	0.0128	0.2193

**Table 7:** The change and the difference in mean changes of LAZ from 12 to 15 months of age as different covariates are added to the mixed-model analysis of covariance.

Model	Adjusted means of LAZ			Difference in mean changes of LAZ			P-values for differences		
	Z-Contr	Z-Suppl	LNS	Z-Suppl *vs* Z-Cont	LNS *vs* Z-Contr	LNS *vs* Z-Suppl	Z-Suppl *vs* Z-Cont	LNS *vs* Z-Cont	LNS *vs* Z-Suppl
Change in LAZ from 12–15 months									
1: No covariates	-0.257 (0.032)	-0.228 (0.020)	-0.073 (0.022)	0.029	0.184	0.155	0.689	< .0001	< .0001
2: Time between measurements only	-0.255 (0.034)	-0.226 (0.024)	-0.075 (0.026)	0.03	0.18	0.151	0.686	0.0003	0.0007
3: Time between measurements, age at midpoint, season	-0.255 (0.033)	-0.252 (0.023)	-0.057 (0.025)	0.004	0.198	0.195	0.994	< .0001	< .0001
4: Above plus covariates at enrollment	-0.257 (0.043)	-0.243 (0.025)	-0.065 (0.028)	0.014	0.192	0.178	0.948	0.004	0.0003
5: Above plus morbidity prevalence and incidence	-0.263 (0.044)	-0.258 (0.026)	-0.049 (0.029)	0.005	0.214	0.209	0.994	0.001	< .0001
6: Above plus LAZ, WAZ and WLZ at start of age-specific period	-0.257 (0.043)	-0.248 (0.027)	-0.058 (0.031)	0.009	0.199	0.191	0.979	0.003	0.0003

Continued breastfeeding was very common in both study areas (>99%), but only a small portion of children met the minimum requirement for adequate dietary diversity [21]. Adding breastfeeding and adequate dietary diversity to the model did not affect the differences in mean changes in LAZ, WAZ and WLZ during any of the age-specific intervals (data not shown).

## Discussion

The present exploratory analyses of change in LAZ and WAZ during age-specific intervals indicated that the supplement type was likely responsible for the significant differences in change in LAZ and WAZ observed from 9 to 12 months and in LAZ from 12 to 15 months between children in the LNS and zinc supplementation trials. Although differences in study duration, seasonality and maternal and child socio-economic characteristics explained some of the differences in study-specific growth patterns, the effect of the supplement type remained significant even after controlling for these other factors. Likewise, the difference in anthropometric outcomes between trials was not explained by differences in morbidity prevalence or incidence. Thus, the present analyses suggest that the difference in study results is likely due at least in part to the fact that children in the LNS study received small-quantity LNS. Considering that the difference in mean change in LAZ occurred when children where 9 to 15 months of age, the results suggest that providing LNS to children at younger age rather than later may result in improved growth, and thus may help prevent stunting [14].

Findings from longitudinal studies investigating the impact of malaria on linear growth are inconsistent [25–29]. Because the malaria prevalence differed between the two study sites in Burkina Faso, we explored whether these illnesses explained the growth differences. However, malaria prevalence and incidence was actually greater in the Dandé Health District, where the LNS study was conducted, and the inclusion of malaria prevalence and incidence into the attenuation model did not affect the observed differences in growth. Thus, malaria was not responsible for the observed growth differences in these studies where malaria surveillance and treatment coverage were very high (>90%).

Several multi-country studies found that diarrhea was associated with a slightly reduced linear growth over the long term [30–32]. Diarrhea prevalence was significantly higher among the LNS study children compared with Z-Suppl in all age intervals. Adding diarrhea prevalence and incidence to the attenuation model did not significantly affect the results for LAZ from 9 to 12 months and 12 to 15 months of age, nor for WAZ from 9 to 12 months of age, indicating that diarrhea was not responsible for the observed study-wise difference in change in growth.

Inclusion of the interval duration between anthropometric measurements and seasonality attenuated the differences in change of growth to a small degree, but the differences remained significant in both the analysis over the whole study period and the age-specific interval analyses. Thus, study design characteristics (i.e. time between anthropometric measurements and seasonality) were not completely responsible for observed growth effects. Similarly, children’s baseline anthropometric status, sex and age only affected growth to a small extent.

Several other studies have also found enhanced growth in response to small-quantity LNS, although results are inconsistent among studies. Adu-Afarwuah and colleagues found a tendency towards improved growth with small-quantity LNS (standardized mean difference of final LAZ of 0.26 between Ghanaian children who received small-quantity LNS from 6 to 12 months compared to children in a non-intervention group), although the difference in final LAZ at 12 months of age was not statistically significant [11]. Small-quantity LNS supplementation for 6 months significantly increased the LAZ (±SE) by 0.13 ± 0.05 in young children in Haiti [12]. This finding is in contrast to a study in Malawi, where the provision of 10, 20 and 40 g of LNS for 12 months to young Malawian children did not prevent growth faltering [9]. An earlier randomized trial in young Malawian children with mean initial LAZ of -1.00 ± 0.77 found that only infants with baseline LAZ below the median who received 50 g LNS gained more length than children who received a micronutrient-fortified maize-soy flour for 12 months [10]. The reasons for these inconsistent growth impacts of small quantity LNS need further investigation.

The strengths and weaknesses of the individual studies included in the present exploratory analyses have been reported previously [14,19]. However, limitations of the present analyses have to be considered. First, for simplicity of interpretation, we assumed for all covariates and outcomes that there were no appreciable group by covariate interactions in the age-specific analysis or study by cohort by covariate interactions in the whole-study analysis. Second, except at the time of initial enrollment into the study, anthropometric assessments at the beginning of any age-specific interval could not be used as covariates, because growth outcomes may have been affected by the prior intervention and therefore show different patterns over time. Thus, we included them in the final models only as mediating variables. Third, other factors not measured in the present analyses may have contributed to the growth impact. In particular, the two studies were implemented in different health districts. Although they were in close geographic proximity, we cannot distinguish between study-related and site-related characteristics. The study fixed effect accounted for part of these site differences in the full study analysis, but was not used in the age-interval analysis because it was confounded with LNS provision. Last, we included all available morbidity results into the analyses independent of treatment, even though treatment coverage differed between the two studies. However, morbidity variables were only weakly related to growth (absolute correlation ranged from 0.002 to 0.106), and therefore including morbidity in the model had little effect. A main limitation was related to the original study designs of the LNS and the zinc supplementation studies. Namely, weekly morbidity surveillance was not done in the NIC, thus we were not able to compare the morbidity burden in the intervention communities and NIC. It is likely that the prevalence of untreated illnesses differed between intervention communities and the NICs, and possible that untreated illnesses had a stronger relationship with growth than treated illnesses. However, for ethical reasons we offered treatment for documented episodes of malaria and diarrhea.

## Conclusions

The present analyses suggest that the difference in growth between the LNS and the zinc supplementation studies is likely at least partly due to small-quantity LNS. Although differences in study design and maternal and child socio-economic characteristics explained some of the study-related differences in observed growth, the effect of the supplement type on LAZ and WAZ remained after controlling for these other factors, and was not explained by differences in morbidity prevalence or incidence. Therefore, the results suggest that providing LNS to children from 9 to 15 months in this setting increases child growth.
